# Supplementary material for: Patterns of Intron Gain and Loss in Fungi
Source: PLoS Biol. 2004 Nov 30;2(12):e422. doi: 10.1371/journal.pbio.0020422 (PMC532390; doi:10.1371/journal.pbio.0020422)
Supplement: Table S1 — Also available at http://genes.mit.edu/NielsenEtAl/. (4.3 MB ZIP). [file pbio.0020422.st001.zip › NielsenEtAl/html/1263.html]

AN4419.1.NCU08158.1.MG09700.1.FG10516.1


```
 CLUSTAL W (1.82) Multiple Sequence Alignments - Introns Inserted


Sequence 1: NCU08158.1	438 aa
Sequence 2: MG09700.1	393 aa
Sequence 3: FG10516.1	385 aa
Sequence 4: AN4419.1	351 aa
Alignment Length: 480 aa
Number Identitical Residues: 94 aa
Alignment Score (without introns) 6126


MG09700.1 	MALSRINGNEELYVGG2VFALRRVQTLEEKNITHIVSVIDYSLEKYQELRG--------K
NCU08158.1	MALNRINGREDLFVGG2IFGVNRARLIEENKITHILSVINYTLPADPAFR---------N
FG10516.1 	MALSRIDGQDELFVGG2LWALRRSDSLTDRRITHVLSLVPFDISSLKNFKDEPWIDYGKD
AN4419.1  	MAMNKVPGYN-IYIGG2VFALKNKVAMSGANITHVLSVIRLR-PKDDLFAG---------
          	**:.:: * : :::** ::.:..   :   .***::*::         : .         

MG09700.1 	FQHMSIDIDDVEDADLLRHFPKLVRFIDGALH------------------------PAGH
NCU08158.1	VQHLSIDIDDVEEADILVHFPKMVRFIERGLYGDEAASIAFGGDNAPVVVPTPASASAKI
FG10516.1 	FRHQLIDIDDVEDSDLLIELPKAVRFIEEGLGAN----------------------YMEH
AN4419.1  	YQHHRIEVDDLDDENLLEHFPSAIKFIQSGLDAG--------------------------
          	 :*  *::**::: ::* .:*. ::**: .* ..                          

MG09700.1 	DDDDQGTSDGD~TDKGAQ----TSPHGSEGGQRGNAVYVHCAMGKSRSVTAVCAYLMHKH
NCU08158.1	NGDDETAHDAD~AITAAPPITTTTITSQQQQTKPGAVLVHCAMGKSRSVSAIVAYLLWKH
FG10516.1 	ADDDGGVSLEK~DVDVVE----------KKRKKKGGVFVHCAVGKSRSVSAVIAYLLRRY
AN4419.1  	----GGVLVHW2PLTASS-----------------------AMGKSRSATVCIAYLLRRQ
          	      .                                  *:*****.:.  ***: : 

MG09700.1 	PARFGA-------ADRSNPD----------RETAARAATQAAVDWVRQTREIAEPNDGFM
NCU08158.1	PHRFGR-------SDPSTP---------------ARRAVTQAINWVRQTRPIAEPNDGFM
FG10516.1 	PSRFDPNITPTAISDPSQSTGSETSAEKRSRKETAQEAVHAALTYVRRTRPMAEPNEGFM
AN4419.1  	RNALTP---------------------------------QSALALLRESRPLCEPNPGFM
          	   :                                     *:  :*.:* :.*** ***

MG09700.1 	KQLALWWEMGTPAD-ADDAVERHPVYQRWLYKREVEESIRIGRAP--DWVRFEDEESAKE
NCU08158.1	EQLELWWTMGCPLESGDDAVENHPAYQRWLYKREVEDATRIGRVP--DWIRFEDEEAAKL
FG10516.1 	EQLALWWEMECPDD-----VESHPVYQRWAYKREIDENLAVGQAP--TRLRFEDEETQPH
AN4419.1  	EQLNVYYQMGCPDD-----VTSHPLYSRWLYRREVEESVACGRAPEMDSVYFEDEQPHQN
          	:** ::: *  * :     *  ** *.** *:**:::    *:.*.   : ****:.   

MG09700.1 	E----DAAATAGPDAQSKVEMRCKKCR~RVLTTQRFIVPHSPAHPTSHKTMPACPHVFVE
NCU08158.1	ASENNNKEAEAGGGAAS-LSLRCKKCR~RTLATKPFIVPHHQGKGNKER---DCGHYFVE
FG10516.1 	D--------DSG------LSLRCKKCR~RTLVTAPFIMEHKPSEKKSSAS--TCQHYFVE
AN4419.1  	V-------ATTG----PATEIKCRKCR2RKLAIAPFVVPHGSHGDVKGAIISECAHIFMS
          	        : :*    .  .::*:*** * *.   *:: *      .    . * * *:.

MG09700.1 	PLSWMRPVLE-----TGELDGRLTCPGAKCGASIGRYSWLGFKCSCGEWVCPAFSLQRSK
NCU08158.1	ALSWMRPTLE-----QGELEGRLTCPNQKCLASVGRYTWQGFRCSCGDWIAPAFSLQKSK
FG10516.1 	PLSWMRGVLE-----KGELNGRLLCPNDKCGAGVGRYDWKGFRCSCGGWVTPAFSLQKAR
AN4419.1  	PLTWMRPSLFPDTPGDAPLSGRLTCPNSSCGSNIGKFAWQGMQCSCGDWVVPAIGLARAR
          	.*:***  * ..:.. . *.*** **. .* :.:*:: * *::**** *: **:.* :::

MG09700.1 	VDEVAVAAT---AARG--ARSGGSDNVEDPRARLGIRMPP----------------~-SL
NCU08158.1	VDEATSAPHPGGAGRGPGGGGGGAAAVAARMAALGIRMPPGGLAGQRGAGEGAGAA~GGP
FG10516.1 	VDDVVKRPT------------GQS---------MGIRMPPG--------------Q~APR
AN4419.1  	VDISEPVTIR--RGPGLPSGIRLPPNMRPIIGAAGVKPESE--------------R2DTA
          	**     .     . .  .    .       .  *::  .                    

MG09700.1 	RENL
NCU08158.1	KENL
FG10516.1 	SGNL
AN4419.1  	KGSL
          	  .*
```
